# Vitamin D and thyroid cancer

**DOI:** 10.1186/1756-6614-6-S2-A28

**Published:** 2013-04-05

**Authors:** Jan Komorowski, Roman Krupiński, Jan Sopiński, Krzysztof Kuzdak, Henryk Stępień, Hanna Ławnicka, Tomasz Stępień

**Affiliations:** 1 Department of Clinical Endocrinology, Medical University of Lodz, Poland; 2 Department of Endocrine and General Surgery, Medical University of Lodz, Poland; 3 Department of Immunoendocrinology, Medical University of Lodz, Poland

## Introduction

The vitamin D system includes a group of fat-soluble pro-hormones and their respective metabolites. Reduced levels of vitamin D_3_ are linked with decrease of calcium and bone homeostasis, the onset and progression of various diseases such as autoimmune diseases, respiratory infections, diabetes mellitus type 1 and type 2, hypertension and cardiovascular disorders, and cancers (breast, colon, liver, stomach and prostate).

This study aimed to investigate vitamin D metabolism by measuring 25(OH)D_3_, 1,25 (OH)_2_D_3_, PTH and calcium concentrations in the peripheral blood of patients with different forms of thyroid tumours.

## Patients and methods

Vitamin D [25(OH)D_3_ and 1,25(OH)_2_D_3_], PTH and calcium serum levels of 50 consecutive patients with epithelial thyroid cancer: 27 cases of papillary cancers (PTC), 16 follicular cancers (FTC), seven cases of anaplastic cancers (ATC), and 34 multinodular nontoxic goiter (MNG) were measured by specific immunoassay. The control group consisted of 26 healthy volunteers.

## Results

The results revealed significantly lower 1,25 (OH)_2_D_3_ concentration in the PTC group (22.67 pg/ml ± 8.12; p <0.05), FTC group (16.09 pg/ml ± 6.15; p <0.02) and ATC group (9.48 pg/ml ± 5.18; p <0.02) versus controls. Levels of 1,25 (OH)_2_D_3_ varied by cancer stage and were also significantly different. A significant decrease in circulating 1,25 (OH)_2_D_3_ concentration was found in patients with stage I (24.12 pg/ml ± 6.77; p <0.05), stage II (16.93 pg/ml ± 4.55; p <0.05), stage III (12.44 ± 8.98; p <0.02) and in stage IVa (6.18 ± 2.22; p <0.01) of cancer. There were no differences when comparing serum levels of 25(OH)D_3_, PTH or calcium concentrations among individuals with multinodular goiter, thyroid cancer and age- and sex-matched control volunteers.

## Conclusions

Our study revealed that impaired vitamin D_3_ metabolism may play an important role in thyroid follicular cell oncogenesis.

